# Case report: Mechanical-electric feedback and atrial fibrillation–Revelation from the treatment of a rare atrial fibrillation caused by annular constrictive pericarditis

**DOI:** 10.3389/fcvm.2023.1100425

**Published:** 2023-01-25

**Authors:** Dong Yi, Lei Li, Min Han, Rujie Qiu, Liang Tao, Li Liu, Chengwei Liu

**Affiliations:** ^1^Division of Cardiac Care Unit, Department of Cardiology, Wuhan Asia Heart Hospital, Wuhan, Hubei, China; ^2^Department of Cardiac Surgery, Wuhan Asia Heart Hospital, Wuhan, Hubei, China

**Keywords:** atrial fibrillation, annular constrictive pericarditis, mechanical-electric feedback, predisposing factors, atrioventricular groove

## Abstract

Atrial fibrillation (AF) is one of the most common arrhythmias encountered in clinical practice. The pathophysiological mechanisms responsible for its development are complex, vary amongst individuals, and associated with predisposing factors. Here, we report a case of AF caused by annular constrictive pericarditis (ACP), which is extremely rare due to its unusual anatomical form. In our patient, AF was refractory to multiple antiarrhythmic medications; however, spontaneous conversion to sinus rhythm occurred when the ring encircling the right and left ventricular (RV and LV) cavities along the atrioventricular (AV) groove was severed. This suggests that atrial stretch due to atrial enlargement and increased left atrial (LA) pressure may contribute to the initiation and maintenance of AF. This report highlights the importance of the careful investigation of rare predisposing factors for AF using non-invasive diagnostic approaches and mechanical-electric feedback (MEF) as a pathophysiological mechanism for AF initiation and maintenance.

## Introduction

Atrial fibrillation (AF) is one of the most common arrhythmias encountered in clinical practice (1, 2). The pathophysiological mechanisms responsible for initiating AF are complex and often vary among individuals (1, 3, 4). Predisposing factors, such as obesity, pericardial fat, obstructive sleep apnea, pre-hypertension, hyperthyroidism, excessive endurance exercise, cardiomyopathies, channelopathies, and heart failure (HF) play an important role in triggering the development of AF (1, 2, 5–9). The regulation of these factors is essential for AF management, such as pharmacological control of HF, however, specific triggers may not be easily identifiable in clinical practice.

Constrictive pericarditis (CP) is a disease characterized by progressive fibrosis, thickening, and/or calcification of the pericardium, which limits heart expansion and leads to symptoms and signs of HF. It may be caused by primary (idiopathic, tuberculosis, posterior viral or bacterial pericarditis, and connective tissue disease-related) or secondary factors (pericardial injury syndrome or radiation therapy) (10, 11). AF was thought to be a part of the natural history of CP; however, it has been reported in only 20–30% of patients with CP (12, 13), and more frequently in those with pericardial calcification (9). Pericardial thickening and/or calcification can be observed in patients with CP using echocardiographic or radiological imaging, although approximately 18% of patients were reported to have a normal pericardium (9). Annular CP (ACP), an extremely rare form of localized CP, is easily missed or misdiagnosed due to its unusual anatomical form. It is a thickened and/or calcified pericardial ring that encircles the right and left ventricular (RV and LV) cavities at the level of the atrioventricular (AV) groove, which leads to cardiac strangulation. As it is very rare and easily missed, AF caused by ACP can become refractory to antiarrhythmic medicines.

Here, we report a case of AF caused by ACP, wherein the diagnosis was complicated, leading to a misdiagnosis for many years. The AF was refractory to multiple antiarrhythmic medications; however, spontaneous conversion to sinus rhythm occurred when the AV groove ring was surgically severed. In this report we highlight the importance of careful investigation of a rare cause of AF and discuss the mechanical-electric feedback (MEF) as a possible pathophysiological mechanism in AF initiation and maintenance.

## Case presentation

A 29-year-old man presented to the hospital with progressively worsening dyspnea and palpitations for 7 years, and intermittent lower extremity edema and abdominal distension for 12 years. He denied chest pain, had no history of hypertension and diabetes, and no family history of heart disease. Personal habits included a small amount of alcohol intake and no smoking. He was diagnosed with bilharziasis, a nephrotic syndrome, approximately 10 years previously, and AF 3 years previously at a local hospital.

**FIGURE 1:**
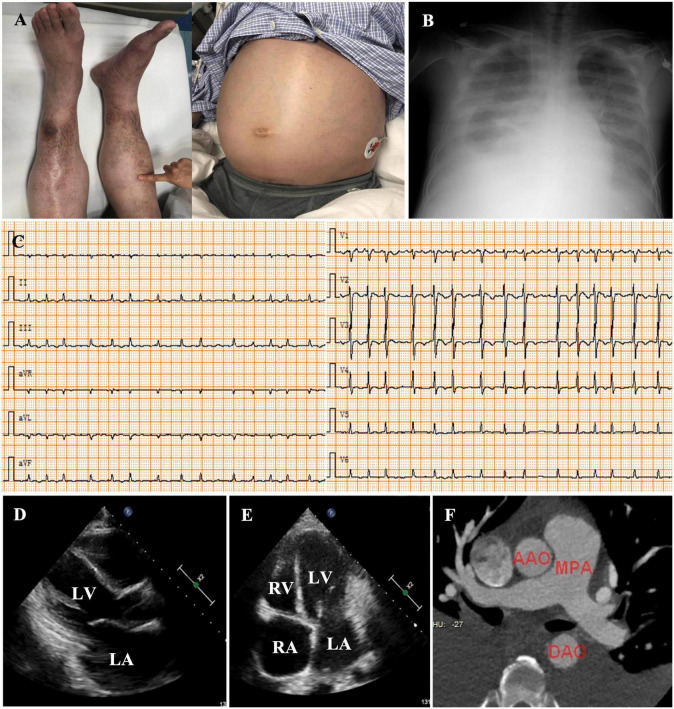
Physical examination revealed pitting edema of the lower extremities and abdominal distension **(A)**. Chest radiology showed a pleural effusion **(B)**. Electrocardiography (ECG) at admission indicated the diagnosis of atrial fibrillation (AF) [heart rate (HR), 134 bpm] with low voltage in all leads **(C)**. Echocardiography showed left **(D)** and RA enlargement **(E)**, and a normal LV chamber. Cardiac computed tomographic angiography of the pulmonary arteries excluded pulmonary embolism **(F)**. LV, left ventricle; RV, right ventricle; LA, left atrium; RA, right atrium; AAO, ascending aortic artery; MPA, main pulmonary artery; DAO, descending aortic artery.

**FIGURE 2:**
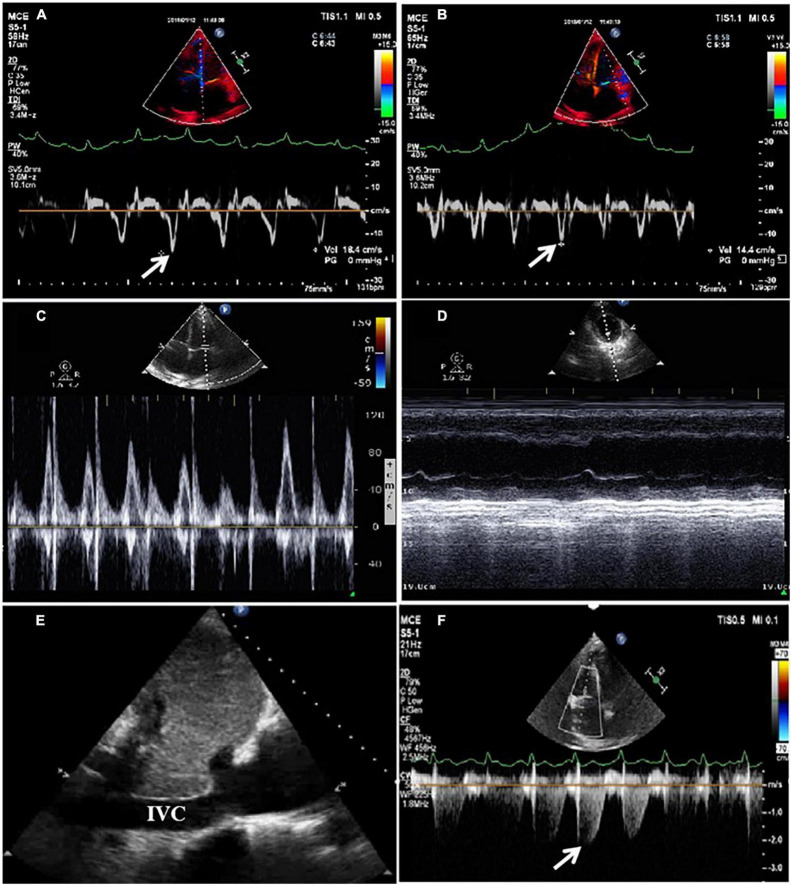
Tissue Doppler imaging (TDI) using echocardiography revealed that e’ = 18.4 m/s (medial) > 14.4 m/s (lateral) in the mitral valve **(A,B)**. Significant respiratory variation of mitral or tricuspid peak E velocity was indeterminable due to atrial fibrillation (AF) **(C)**. Paradoxical septal motion during respiration was not observed **(D)**. The inferior vena cava (IVC) was dilated without inspiratory collapse **(E)**. Pulmonary artery pressure was elevated according to the velocity of tricuspid regurgitation **(F)**.

**FIGURE 3:**
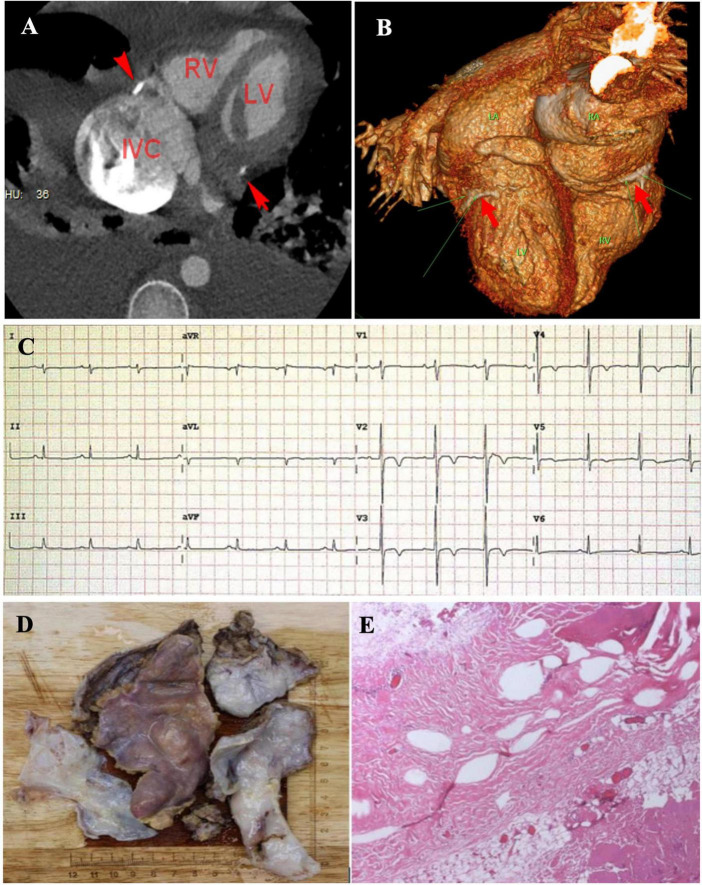
Cardiac computed tomographic angiogram of four-chamber view **(A)** and volume rendering views **(B)** showed calcified annular constrictive pericarditis (ACP) trapping both ventricles (red arrows). Spontaneous conversion from atrial fibrillation (AF) to sinus rhythm as the calcified pericardial ring was severed **(C)**. Complete pericardiectomy was performed and revealed a thickened pericardium with calcification embedded in the atrioventricular (AV) groove **(D)**. Pathological examination of the pericardial tissue indicated fibrotic tissue with calcification **(E)**.

On admission, he was hypotensive [blood pressure (BP), 95/60 mmHg] and weak. A physical examination revealed distended jugular veins, abdominal distension, pitting edema of both lower extremities (Figure 1A), decreased breath sounds in the right lower lung, and a grade II/VI systolic murmur at the apex of the heart. Routine blood, renal function, plasma electrolyte concentration, troponin, and inflammatory and tumor marker tests were normal. He had mild liver dysfunction and elevated D-dimer (19.20 g/ml) and plasma B-type natriuretic peptide (NT-proBNP, 1,987 pg/ml). Chest radiology revealed a right-sided pleural effusion and cardiac enlargement (Figure 1B). AF [heart rate (HR), 154 bpm] without ST-segment and T-wave changes was confirmed on electrocardiography (ECG) (Figure 1C). Transthoracic echocardiography (TTE) revealed left atrial (LA) and right atrial (RA) enlargement (LA, 51 mm; RA, 53 mm), mild mitral and tricuspid regurgitation, decreased LV systolic function (LVEF, 45%), diastolic dysfunction, and a normal LV size (42 mm) (Figures 1D, E). Abdominal ultrasonography revealed a large amount of abdominal fluid, liver congestion, and inferior vena cava (IVC) distention. Pleural and abdominal effusion analyses indicated transudative fluid. Tuberculosis and rheumatism were excluded based on laboratory test results and effusion fluid analysis. Pulmonary embolism was excluded using computed tomography angiogram (CTA) (Figure 1F). Chemical cardioversion was attempted unsuccessfully using several antiarrhythmic medications (amiodarone and nifekalant). A repeat TTE revealed mitral lateral annulus e’ velocity (14.4 cm/s) < mitral medial annulus e’ velocity (18.4 cm/s) using tissue Doppler imaging (TDI) (Figures 2A, B). We were unable to determine significant respiratory variation of mitral and tricuspid peak E velocity due to the AF (Figure 2C). Paradoxical septal motion during respiration was not observed (Figure 2D). The IVC was dilated without inspiratory collapse (Figure 2E). The pulmonary artery pressure (∼30 mmHg) was elevated according to the velocity of the tricuspid regurgitation (Figure 2F). Careful review of the echocardiographic images showed nodular thickening of the pericardium in the AV groove, without significant thickening of the whole pericardium. We then measured the peripheral venous pressure, which was elevated (43 cm H_2_O). These findings supported but did not confirm the diagnosis of CP. ACP was diagnosed using further CT scan analysis, which showed a thickened and calcified pericardial ring around each AV groove with minimal pericardial calcification; however, radiological pericardial thickening and calcification were not observed on chest X-ray (CXR) (Figures 3A, B). Intraoperatively, a calcified pericardial ring encircling the RV and LV cavities at the level of the AV groove was revealed. As the calcified pericardial ring was severed, spontaneous conversion to sinus rhythm was observed (Figure 3C). Pathological examination of the pericardial tissue revealed pericardial thickening, fibrous tissue hyperplasia, and hyaline degeneration, in which a large number of capillaries and few inflammatory cells were observed. A large amount of fat was observed on the surface, confirming the diagnosis of ACP (Figures 3D, E). Post-operatively, the patient’s symptoms and vital signs improved significantly. The lower extremity edema, pleural effusion, and ascites subsequently subsided, and the pulmonary congestion and cardiac enlargement mitigated, indirectly indicating a reduced constrictive pattern. The patient was followed up on an outpatient basis and remained in good clinical condition. The echocardiographic assessment at the 1-month follow-up showed that the enlarged atria became smaller (LA, 39 mm; RA, 38 mm) and LV ejection fraction (LVEF) significantly improved (from 45% pre-operation to 57%). The patient management timeline during hospitalization is summarized in Table 1. This study was approved by the local Ethics Committee, and written informed consent was obtained from the patient for the publication of any potentially identifiable images or data included in this article.

**TABLE 1:** The timeline of patient’s management during hospitalization.

Timeline of the case management	
Day 1	● Admission ● Lab tests ● Management with medications
Day 2	● Chest X-ray ● Echocardiography
Day 3	● CTA ● Diagnostic thoracocentesis and abdominocentesis and fluid analysis
Day 4	● Repeated Echocardiography and Color Doppler ● Review results of lab test, echocardiography, radiological Imaging
Day 5	● Heart team consultation including cardiac surgeons ● Reached the diagnosis of ACP
Day 7	● Pericardiectomy ● Transferred to ICU
Day 12	● Discharge

CTA, computed tomographic angiogram; ACP, annular constrictive pericarditis; ICU, intensive care unit.

## Discussion

Annular constrictive pericarditis is an extremely rare form of localized CP. Anatomically, a thickening fibrous band, with or without calcification, encircles the right and left cavities at the level of the AV groove, and strangulates the heart. HF symptoms, such as exertional dyspnea and edema, are commonly reported by patients with ACP (10, 14). The diagnosis is easily missed due to the absence of a thickened/calcified pericardium, which is commonly seen in CP. TTE may reveal some characteristics of cardiac constriction, such as interventricular dependence, changes in mitral TDI velocity, respiratory variation in transmitral flow, and dilation of the IVC without inspiratory collapse (10, 15); however, these characteristics are non-specific and seen in other disorders, such as cardiomyopathy. In our patient, TTE revealed a normal LV size and systolic function, paradoxical septal motion during respiration was not observed, and the respiratory variation of mitral and tricuspid peak E velocity was indeterminable due to AF, although bi-atrial enlargement and dilation of the IVC were observed. Due to the rarity and anatomical form of ACP, careful examination of the AV groove was required to make a diagnosis; therefore, radiological imaging was another useful diagnostic modality for detecting pericardial thickening and/or calcification. In our patient, a thickened/calcified band in the AV groove was initially missed and ACP was diagnosed using further CT scan analysis based on suspicion, and the diagnosis was confirmed surgically. Cardiac catheterization is the gold standard for diagnosing CP, and can be used to differentiate it from other diseases with restrictive physiology. Typically, elevation and equalization of diastolic pressures in all four chambers can be observed. The RV and LV waveforms exhibit a “dip and plateau (or square root)” sign (16). However, we did not perform cardiac catheterization prior to surgery due to the patient’s condition (very weak), which could have provided diagnostic evidence of CP.

The precise pathophysiological mechanisms of AF remain elusive; however, the following two different mechanisms have been proposed: abnormal impulse formation and reentrant activity. Most sustained atrial arrhythmias have been ascribed to the reentrant mechanism (17). AF frequently occurs in patients with atrial dilatation (18, 19). Atrial dilatation and/or elevated atrial pressure induces local or global changes in cardiac electrophysiology by increasing the atrial surface, shortening the refractory period, and/or slowing the conduction velocity (20, 21). This phenomenon suggests the role of MEF in atrial arrhythmogenesis. Both experimental and clinical studies have confirmed that atrial stretch induces the onset of atrial arrhythmias through the modulation of myocardial electrophysiological properties (i.e., refractory period (RP) and conduction velocity) (21, 22). In contrast to CP, which limits expansion and filling of all four chambers, ACP mainly affects atrial structure and function through strangling the outflow tract of the atria, leading to atrial dilation and dysfunction. In such situations, drug-therapy is usually ineffective if the mechanical stretch is unresolved. The spontaneous conversion from AF to sinus rhythm and the resection of the annular ring in our patient confirmed this opinion, suggesting the role of MEF as a possible pathophysiological mechanism in ACP -induced AF.

Annular constrictive pericarditis is an extremely rare form of localized CP, which is easily missed or misdiagnosed due to its unusual anatomical form. Careful examination of the AV groove using radiological imaging should be performed in patients with clinical suspicion of underlying CP, without evidence of pericardial thickening. ACP-induced AF is usually refractory to antiarrhythmic medications and surgical resection of the connective ring is imperative. MEF was a possible pathophysiological mechanism in ACP-induced AF.

## Data availability statement

The original contributions presented in this study are included in the article/supplementary material, further inquiries can be directed to the corresponding authors.

## Ethics statement

Written informed consent was obtained from the patient for the publication of any potentially identifiable images or data included in this article.

## Author contributions

DY, LeL, MH, RQ, LT, LiL, and CL contributed to the patient diagnosis, treatment, and follow-up. DY and LeL drafted this manuscript. LiL and CL revised the final version of the manuscript. All authors agreed to be accountable for the content of the work and approved the submitted version.
